# New mechanisms of antiplatelet activity of nifedipine, an L-type calcium channel blocker

**DOI:** 10.7603/s40681-014-0024-z

**Published:** 2014-11-18

**Authors:** Tz-Chong Chou

**Affiliations:** 1 Institute of Medical Sciences, Tzu Chi University, 6F, Xie-Li Building, No. 707, Sec. 3, Zhongyang Rd.,, 970 Hualien, Taiwan; 2 Department of Life Sciences, Tzu Chi University, 970 Hualien, Taiwan; 3 Department of Biotechnology, Asia University, 413 Taichung, Taiwan

**Keywords:** Nifedipine, Peroxisome, proliferator-activated, receptors, Nuclear factor-κB, Platelet aggregation, Protein kinase Cα, Nitric oxide

## Abstract

Platelet hyperactivity often occursd in hypertensive patients and is a key factor in the development of cardiovascular diseases including thrombosis and atherosclerosis. Nifedipine, an L-type calcium channel blocker, is widely used for hypertension and coronary heart disease therapy. In addition, nifedipine is known to exhibit an antiplatelet activity, but the underlying mechanisms involved remain unclear. Several transcription factors such as peroxisome proliferator-activated receptors (PPARs) and nuclear factor kappa B (NF-κB) exist in platelets and have an ability to regulate platelet aggregation through a non-genomic mechanism. The present article focuses on describing the mechanisms of the antiplatelet activity of nifedipine *via* PPAR activation. It has been demonstrated that nifedipine treatment increases the activity and intracellular amount of PPAR-β/-γ in activated platelets. Moreover, the antiplatelet activity of nifedipine is mediated by PPAR-β/-γ-dependent upon the up-regulation of the PI3K/AKT/NO/cyclic GMP/PKG pathway, and inhibition of protein kinase Cα (PKCα) activity *via* an interaction between PPAR-β/-γ and PKCα. Furthermore, suppressing NF-κB activation by nifedipine through enhanced association of PPAR-β/-γ with NF-κB has also been observed in collagen-stimulated platelets. Blocking PPAR-β/-γ activity or increasing NF-κB activation greatly reverses the antiplatelet activity and inhibition of intracellular Ca^2+^ mobilization, PKCα activity, and surface glycoprotein IIb/IIIa expression caused by nifedipine. Thus, PPAR-β/-γ- dependent suppression of NF-κB activation also contributes to the antiplatelet activity of nifedipine. Consistently, administration of nifedipine markedly reduces fluorescein sodium-induced vessel thrombus formation in mice, which is considerably inhibited when the PPAR-β/-γ antagonists are administrated simultaneously. Collectively, these results provide important information regarding the mechanism by which nifedipine inhibits platelet aggregation and thrombus formation through activation of PPAR-β/-γ- mediated signaling pathways. These findings highlight that PPARs are novel therapeutic targets for preventing and treating platelet-hyperactivity-related vascular diseases.

## Abstract

Platelet hyperactivity often occursd in hypertensive patients and is a key factor in the development of cardiovascular diseases including thrombosis and atherosclerosis. Nifedipine, an L-type calcium channel blocker, is widely used for hypertension and coronary heart disease therapy. In addition, nifedipine is known to exhibit an antiplatelet activity, but the underlying mechanisms involved remain unclear. Several transcription factors such as peroxisome proliferator-activated receptors (PPARs) and nuclear factor kappa B (NF-κB) exist in platelets and have an ability to regulate platelet aggregation through a non-genomic mechanism. The present article focuses on describing the mechanisms of the antiplatelet activity of nifedipine *via* PPAR activation. It has been demonstrated that nifedipine treatment increases the activity and intracellular amount of PPAR-β/-γ in activated platelets. Moreover, the antiplatelet activity of nifedipine is mediated by PPAR-β/-γ-dependent upon the up-regulation of the PI_3_K/AKT/NO/cyclic GMP/PKG pathway, and inhibition of protein kinase Cα (PKCα) activity *via* an interaction between PPAR-β/-γ and PKCα. Furthermore, suppressing NF-κB activation by nifedipine through enhanced association of PPAR-β/-γ with NF-κB has also been observed in collagen-stimulated platelets. Blocking PPAR-β/-γ activity or increasing NF-κB activation greatly reverses the antiplatelet activity and inhibition of intracellular Ca^2+^ mobilization, PKCα activity, and surface glycoprotein IIb/IIIa expression caused by nifedipine. Thus, PPAR-β/-γ- dependent suppression of NF-κB activation also contributes to the antiplatelet activity of nifedipine. Consistently, administration of nifedipine markedly reduces fluorescein sodium-induced vessel thrombus formation in mice, which is considerably inhibited when the PPAR-β/-γ antagonists are administrated simultaneously. Collectively, these results provide important information regarding the mechanism by which nifedipine inhibits platelet aggregation and thrombus formation through activation of PPAR-β/-γ- mediated signaling pathways. These findings highlight that PPARs are novel therapeutic targets for preventing and treating platelet-hyperactivity-related vascular diseases.

## Introduction

Platelets are unnucleated fragments derived from bone marrow megakaryocytes. Traditionally, the most well-known function of platelets is that they are responsible for hemostasis in response to vascular injury and endothelial disruption. Recent studies have indicated that platelets also have an immunomodulatory activity through production of several pro-inflammatory mediators promoting pathogenic thrombi formation and inflammatory responses [1, 2]. Platelets perform their functions mainly through secretion of several proteins stored in various cytoplasmic granules. There are at least three different types of granules (α-granules, dense core granules, lysosomes), and a complex membranous system in platelets. The α-granules contain hemostatic factors (factor V, von Willebrand factor (vWF) and fibrinogen) and other cytokines, mitogenic factors (PDGF and bFGF) and proteases (MMP2, MMP9) [3]. The mediators stored in α-granules can be selectively released in response to the activation of different receptors. Dense granules store small non-protein molecules such as ADP, ATP, serotonin, calcium and pyrophosphate, which all play a central role in the amplification of platelet aggregation. Lysosomes contain glycosidases, proteases, and cationic proteins with bactericidal activity.

Excessive platelet activation has been regarded as a key pathological factor in the development of many vascular diseases such as acute coronary syndromes, myocardial infarction and atherothrombosis [4, 5]. Endothelial dysfunction/injury initially induces platelet activation, and promoting their interaction with neutrophils and monocytes leads to the pathogenesis of atherosclerosis. Therefore, platelets are an important link between tissue damage and hemostatic and inflammatory responses. In supporting this concept, several lines of evidence have demonstrated that platelet hyperactivity often occurrs in hypertensive or cardiovascular patients [6, 7]. Thus, agents with inhibiting platelet hyperactivity may be potential therapeutic drugs for platelet-related vascular diseases.

**Fig. 1:**
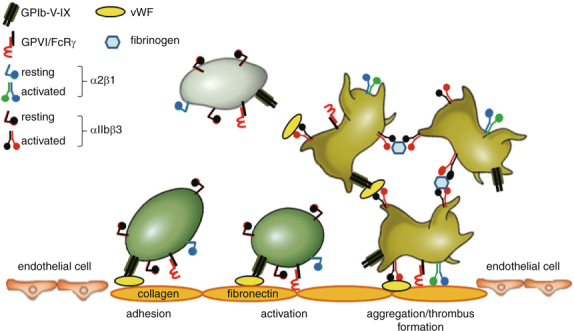
Platelet adhesion and aggregation at the sites of vascular injury. The interaction of GPIb-V-IX and vWF enables GPVI binding to collagen in injured vessels. This triggers platelet aggregation by crosslinking adjacent platelets through binding to fibrinogen and vWF *via* the activated GPIIb–IIIa receptors.

## Platelet activation

Platelet adhesion to the extracellular matrix is the initial step in haemostasis [8]. When vascularity is damaged, the immobilized vWF on exposed collagen becomes a strong adhesive substrate. The vWF, a multimeric adhesive glycoprotein, contains binding sites for collagen glycoprotein (GP)Ib and integrin GPIIb/IIIa (αIIbβ3) [9]. The adhesion is mediated by the interaction between the GPIb-IX-V receptor complex on the platelet surface to vWF, and GPVI and GPIα to collagen at sites of vascular injury. The interaction of vWF and GPIb-IX-V complex is required for the adhesion of platelets to the subendothelium, which enables GPVI binding to collagen [10]. In addition, collagen serves as a binding site for vWF in the subendothelial matrix, and therefore contributes to the adhesion of unactivated platelets *via* GPIb-IX-V (Figure 1) [11]. The adhesion is followed by platelet aggregation by binding to soluble fibrinogen and vWF *via* the activated integrin GPIIb/ IIIa. Collectively, upon activation of the glycoprotein receptors, it promotes platelet adhesion, aggregation, and spreading on the exposed extracellular matrix of the injured vessel wall, as well as thrombus formation and stability [12].

There are multiple pathways regulating platelet activation. The platelet agonists including ADP, thrombin, collagen and serotonin perform their functions through their specific receptors of platelets. ADP stored in dense granules is released during platelet activation. ADP promotes platelet activation through its receptors (P2Y_1_ and P2Y_12_). P2Y_1_ is a G-protein-coupled seven-transmembrane domain receptor that stimulates platelet shape change and mobilizes calcium from intracellular stores by activating phospholipase C (PLC) [13]. Activation of the P2Y_12_ receptor inhibits the adenylate cyclase activity of platelets and seems to be responsible for a positive feedback mechanism for platelet stimulation especially by weak agonists [14]. There are two receptors (protease activated receptors 1 (PAR1) and 4 (PAR4)) on platelet surfaces for thrombin, the most potent physiological platelet activator [15]. PAR1 mediates platelet activation at low concentrations of thrombin, while PAR4 is activated at higher thrombin concentration. PARs are also expressed in other cells in the vasculature, cells such as leukocytes, endothelial cells and smooth muscle cells. It has been reported that PAR-1 and PAR-2 expressed in the vessel wall are involved in contractility, inflammation, proliferation, and repair [16]. Collagen is also a strong platelet activating agent because it stimulates platelet adhesion and is mediated by the binding of vWF to it at the sites of vascular injuries. Moreover, other glycoprotein receptors such as GPIb, GPIIb/IIIa, GPIa/IIa, and GPVI all contribute to collagen-mediated platelet activation [1]. Serotonin expresses its actions through the serotonin receptor 5-HT2A to enhance procoagulant activity *via* retention of fibrinogen and thrombospondin on platelet surfaces. Furthermore, epinephrin and other catecholamines stimulate the platelet α2Aadrenergic receptor, which coupled with a G-protein leads to the inhibition of adenylate cyclase activity, which in turn induces platelet aggregation [17].

When platelets are activated by collagen or thrombin, arachidonic acid (AA) is liberated from membrane phospholipids. This liberation is caused by Ca^2+^-dependent phospholipase A_2_ (PLA_2_), diglyceride lipase and/or phosphatidic acid specific phospholipase A_2_ [18]. Then, AA is converted to thromboxane A_2_ (TXA_2_), a potent activator for the release reaction and aggregation of platelets by cyclooxygenase (COX) and thromboxane synthase. TXA_2_, when released from the platelets, binds to the G-protein coupled thromboxane receptor and functions as an agonist for platelet activation [19]. In addition, TXA_2_ also participates in a positive feedback loop further increasing [Ca^2+^]_i_ and AA-induced ATP release and platelet aggregation [20]. Phosphoinositides breakdown is another critical mechanism accounting for agonist-induced platelet activation. Simulation by agonists such as thrombin or collagen results in PLC-catalyzed hydrolysis of plasma membrane phospholipid phosphatidylinositol 4,5-bisphosphate, with concomitant formation of inositol 1,4,5,-trisphosphate (IP_3_) and diacylglycerol [21]. The IP_3_ binds to the IP_3_ receptor on sarcoplasmatic reticulum (SR) triggering a significant release of Ca^2+^ from SR, leading to an increase of intracellular Ca^2+^ concentration [22]. It has been demonstrated that an increase of intracellular Ca^2+^ concentration, as a result of either calcium influx and/or calcium release from intracellular stores, is fundamental to platelet activation [23]. Importantly, diacylglycerol acts as an activator for protein kinase C (PKC) that is essential for agonist-induced platelet aggregation and granule secretion [24]. At least seven PKC isoforms (α, β, δ, θ, ε, η, and ζ) are found in platelets. Among these PKC isoforms, activation of PKCα through Syk-dependent phosphorylation is crucial for platelet activation [25]. Moreover, TXA_2_ has an ability to activate PKC by activating PLC-dependent pathways [26], suggesting that blocking TXA_2_ formation may inhibit PKCα-related signaling pathways. Mitogen-activated protein kinases (MAPKs) containing extracellular signal-regulated kinase (ERKs), the c-Jun N-terminal kinase (JNK) and p38 MAPK have been identified in platelets [27]. The roles of JNKs and ERKs in platelet mechanisms are still unclear. On the other hand, activation of p38 MAPK by PKC can phosphorylate cPLA2 on Ser505, leading to the production of AA and TXA_2_ synthesis [28]. Thus, cPLA_2_ activation is regulated, at least in part, by PKC through p38 MAPK. These platelet-activating mechanisms ultimately up-regulate surface GPIIb/IIIa expression, thereby promoting the binding of fibrinogen and platelet aggregation.

## Antiplatelet mechanisms

### NO/cyclic GMP

Nitric oxide (NO), synthesized from L-arginine by nitric oxide synthase (NOS), activates intracellular soluble guanylyl cyclase (sGC) and guanosine 3’,5’-cyclic monophosphate (cGMP) formation, which subsequently activates cGMP-dependent protein kinase (PKG). The NO-dependent signaling pathway is known to play an important modulatory role both in physiological and pathological conditions [29]. Up-regulation of the NO/cGMP/ PKG1 cascade reportedly inhibits platelet activation by regulating actin filament dynamics, integrin activation, and intracellular Ca^2+^ mobilization, which in turn suppresses PLC and PKC activity [30, 31]. Moreover, PKG promotes sarcoplasmic reticulum ATPase (SERCA)-dependent refilling of intraplatelet Ca^2+^ stores and inhibits inositol-1,4,5-trisphosphate-stimulated Ca^2+^ release from the sarcoplasmic reticulum, which in turn decreases intracellular Ca^2+^ level and platelet activation [30]. PKG is capable of phosphorylating the TxA_2_ receptor, thereby inhibiting its function. cGMP also indirectly increases intracellular cAMP through inhibition of phosphodiesterase type 3 to synergistically inhibit platelet aggregation [33]. Interestingly, previous studies have indicated that the actions of NO on platelet function are also mediated by a cGMP-independent mechanism that inhibits exocytosis of platelet granules (dense, lysosomal, and α-granules) by S-nitrosylation of N-ethylmaleimidesensitive factor (NSF) [34].

### Cyclic AMP

It has been demonstrated that the elevation of cyclic AMP formation reduces platelet functions including adhesion, aggregation, the release of granule contents as well as the rise of intracellular Ca^2+^ mobilization [33, 35]. The steady-state level of cyclic AMP is maintained by a balance between the rate of synthesis by adenylate cyclase and the rate of degradation by cyclic AMP phosphodiesterase. Research has shown that several cyclic AMP-elevating agents exert antiplatelet activity through a cyclic AMP-dependent protein kinase (PKA)-dependent signal pathway [36, 37].

### GP IIb/IIIa Inhibitors

When platelets are activated by physiological agonists such as thrombin, ADP, or collagen, the intracellular signal pathways for platelet activation are stimulated and thereby induce conformational changes of αIIbβ3 leading to the formation of an activated state with high affinity for fibrinogen and numerous other ligands [1, 38]. As a result of the enhancement of αIIbβ3- mediated binding to the bivalent molecule, fibrinogen may cause platelets aggregation. To date, two binding sites have been well characterized in αIIbβ3: an Arg-Gly-Asp (RGD)-binding site and a Lys-Glu-Ala-Gly-Asp-Val (KQAGDV)-binding site. Fibrinogen binds *via* the KQAGDV-binding site. Agents that bind within the ligand-binding region of αIIbβ3 and block the binding of its natural ligands have been developed and termed GPIIb/IIIa inhibitors. There are three FDA-approved integrin αIIbβ3 inhibitors, and they include abciximab (ReoPro; Lilly), eptifibatide (Integrilin; millennium Pharmaceuticals/Schering–Plough), and tirofiban (Aggrastat; merck). Abciximab is a murine human chimeric fab fragment that was derived from the murine monoclonal antibody 7E3. Eptifibatide is a KGD-containing cyclic heptapeptide. And tirofiban is a non-peptide derivative based on the RGD sequence [39]. Clinical studies have indicated that a blockade of the glycoprotein IIb/IIIa receptors limits the inflammatory responses secondary to coronary intervention, suggesting that inhibition of inflammatory marker expression by GPIIb/IIIa inhibitors may contribute to its clinical benefit [40]. Our previous study tested the effect of the synthesized RGRHGD with the highest local hydrophilicity region of B chain of β-bungarotoxin on platelet aggregation. The RGRHGD holds parts of both RGD and KGD peptides that have been reported to exhibit a high binding affinity to GPIIb/IIIa. Moreover, the inhibitory effect of RGRHGD on platelet aggregation is associated with attenuation of TXA2 formation and intracellular calcium mobilization. These findings may, at a later date, provide a useful method for finding potential therapeutic agents through molecular modeling analysis [41].

## Peroxisome proliferator-activated receptors (PPARs) and platelet activation

PPARs belonging to ligand-activated transcription factors modulate several important biological effects, including lipid, glucose homeostasis, energy metabolism, and inflammation [42, 43]. A variety of compounds can serve as PPAR ligands and activate the receptor. PPARα ligands are fatty acids, and their derivatives as well as eicosanoids include 8-S-hydroxyeicosatetraenoic acid (8SHETE) and leukotriene B4 (LTB4) [44]. Moreover, fibrates, the synthetic ligands for PPARα, are widely used in the treatment of hypertriglyceridemia and hyperlipidemia [44]. Other pharmacological compounds such as nonsteroidal anti-inflammatory drugs (NSAIDs) are confirmed as PPARα ligands [45]. PPARγ is activated by 15-deoxy-12, 14-prostaglandin J2 (PG-J2) and 15- hydroxyeicosatetraenoic acid (15-HETE) [46, 47] that are AA metabolites derived from COX and lipoxygenase pathways. In addition, fatty acid-derived compounds of oxidised LDL, including 9- and 13-hydroxyoctadecadienoic acid (9- and 13-HODE), and glitazones [48] (an antidiabetic drug), indomethacin, and ibuprofen all function as ligands for PPARγ [45]. In response to their ligands, PPARs undergo a conformational change leading to the recruitment of distinct coactivators and corepressors. Subsequently, these changes result in PPAR heterodimerization with cis-retinoid X receptor (RXR) and in turn regulate downstream gene expression by binding to the peroxisome proliferator response element (PPRE) that exist in the promoter of target genes of nucleated cells. Although platelets are anuclear cells, they also contain transcription factors such as PPARs. The existence of three PPAR isoforms (α, β/δ, and γ) in human platelets has been demonstrated, and activation of PPARs inhibits platelet aggregation through a nongenomic mechanism [49, 50]. It has been reported that the inhibitory effect of PPAR agonists on platelet aggregation is associated with the modulation of GPVI, PKCα and calcium mobilization signals [51]. Our recent study indicated that the antiplatelet activity of alpha-lipoic acid is mediated by PPAR-α/-γ-dependent processes [50]. Therefore, reagents exerting PPAR-activating activity have been regarded as a new class of antiplatelet drugs.

## PPARs and atherosclerosis

Atherosclerosis is a complex process characterized by lipid accumulation in the arterial wall resulting in heart and brain infarction. The atherogenesis is initiated *via* the attraction of various cells such as monocyte/macrophages, T lymphocytes, endothelial cells, and smooth muscle cells (SMCs). This cellular activation promotes local inflammatory responses and migration and proliferation of SMCs, which in turn leads to the formation of foam cells. PPARs are expressed in the vascular wall and atherosclerotic lesions, suggesting that they may modulate the atherogenic processes. Clinical studies have indicated that PPARγ ligand troglitazone inhibits SMC proliferation and decreases the intima and media thickness of carotid arteries [52]. The inhibitory effects of PPARs (α and γ) on the expression of inflammatory genes, such as interleukin-6, cyclooxygenase-2, inducible nitric oxide synthase (iNOS), matrix metalloproteinase-9, endothelin-1, lipid accumulation within the plaque, and thrombogenesis [53] have been proposed to be the underlying mechanisms for their anti-atherosclerotic effects. Furthermore, PPARα activators also can induce apoptosis of activated macrophages by inhibiting the antiapoptotic NF-κB pathway [54] and reducing monocytic recruitment to early atherosclerotic lesions by inhibition of monocyte-recruiting proteins such as vascular cell adhesion molecules (VCAM)-1 expression in endothelial cells [55]. These findings suggest that up-regulation of PPAR expression/activation may prevent the progress of atherosclerotic disease.

## Nifedipine, a dihydropyridine calcium channel blocker (CCB)

The calcium channel blockers (CCB) are a group of drugs used to treat cardiovascular diseases including hypertension, angina, and peripheral vascular disorders. CCBs were approved for the treatment of hypertension in the 1980s. Since then, CCBs have increased markedly because of their effective lowering of blood pressure with few side effects. In addition to the cardiovascular effects of CCBS, other beneficial functions of CCBs, such as antioxidative, anti-inflammatory, anti-atherosclerotic, bone-remodeling, and immunomodulating properties have been reported [56]. It is well known that inflammation is a fundamental basis of atherosclerosis. Several reports have revealed that dihydropyridine CCBs exert anti-inflammatory effect by suppressing the tumor necrosis factor (TNF), monocyte chemoatractant protein-1 (MCP-1), and pro-inflammatory cytokine expression accompanied by the reduction of NF-κB activation in various vascular cells including endothelial cells, macrophages, and smooth muscle cells [56]. These actions of CCBs may contribute to the anti-atherosclerotic effect in vascular cells.

CCBs can be classified into 3 main classes according to their different structure. The three classes are the phenylalkylamines (e.g., verapamil), the benzothiazepines (e.g., diltiazem), and the dihydropyridines (e.g., nifedipine, amlodipine, isradipine). It is known that different classes of CCBs have differing pharmacologic actions. With their relative potency of lowering blood pressure, the dihydropyridine-type compounds such as nifedipine are the most potent subclass. Nifedipine, a dihydropyridine-based L-type CCB, is widely used in the treatment of hypertension and coronary heart diseases. Clinical studies have shown that a significant reduction of new coronary lesions and the intima-media thickness in the carotid artery were observed in patients treated with nifedipine [57]. These findings confirmed that nifedipine has an anti-atherosclerotic effect beyond its blood pressure-lowering effect. The protection may be associated with suppressing reactive oxygen species (ROS) formation and subsequent inflammatory responses, as well as smooth muscle cell proliferation, migration and differentiation [56]. Importantly, nifedipine can activate PPAR-γ by inhibiting ERK1/2 activity in macrophages [58], suggesting that PPARs may involve the pharmacological effects of nifedipine. As increased intracellular Ca^2+^ concentration ([Ca^2+^]_i_) is essential for platelet activation, therefore, reagents that attenuate platelet [Ca^2+^]_i_ may have an antiplatelet activity. As expected, nifedipine is capable of inhibiting platelet aggregation, though platelets lack L-type calcium channels. To date, the underlying molecular mechanisms remain unclear; as although the activation of NO/cGMP-dependent signaling pathway [59, 60] has been proposed as a possible mechanism that contributes to its antiplatelet activity of CCBs.

## Nifedipine-mediated anti-aggregatory effect *via* activation of PPAR-β/-γ

PPARs play an important role in the modulation of metabolism and inflammatory processes, and exhibit a protective effect against the development of atherosclerosis and cardiovascular diseases [61, 62]. Traditionally, the actions of transcription factors are thought to be mainly attributed to their regulation in gene expression. Recently, accumulating evidence supports that there are nongenomic actions of these receptors [63]. Because a number of transcription factors including PPARs, estrogen receptors (ER), and nuclear factor kappa B (NF-κB) have been found in platelets, many studies have focused on the role of cytoplasmic PPARs in platelet function. It has been reported that the activation of PPARs (α, β/δ, and γ) by respective agonists inhibits platelet aggregation [49, 50] and slows intraarterial thrombus formation due to increased NOS expression [64]. Thus, selective ligands for PPARs may negatively regulate platelet activation. Based on the finding that nifedipine induces PPAR-γ activation in macrophages and smooth muscle cells [58], it is possible that PPARs may involve nifedipine-mediated regulation of platelet and vascular functions.

**Fig. 2:**
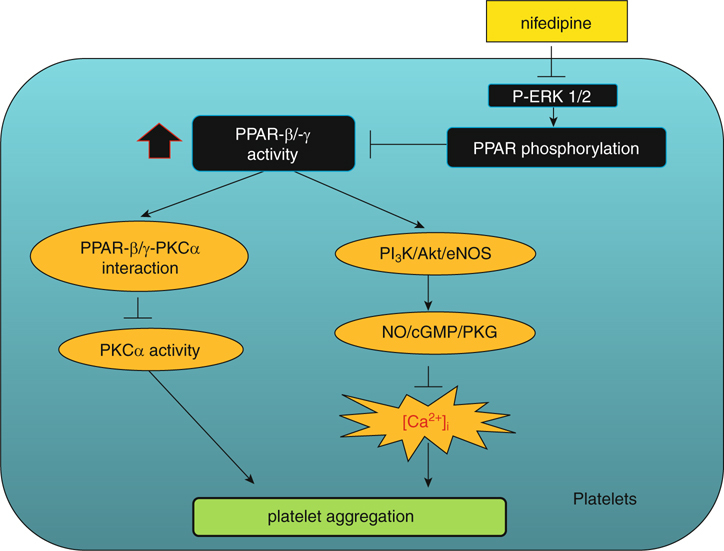
The antiplatelet activity of nifedipine is mediated by PPAR-β/γ. Nifedipine increases the activity and intracellular levels of PPAR-β/γ in activated platelets. Subsequently, PPAR-β/-γ-dependent up-regulation of PI_3_K/Akt/eNOS/NO/cyclic GMP/PKG cascade, inhibition of PKCα activity *via* association of PPAR-β/γ with PKCα, and intracellular Ca^2+^ mobilization ultimately inhibits platelet aggregation.

Our recent study has confirmed that treatment with nifedipine significantly increases PPAR-β and PPAR-γ activity due to the inhibition of phosphorylation of ERK1/2 and PPAR-γ without affecting PPAR-α activity in collagen-stimulated platelets [65]. These results indicat that nifedipine is a dual PPAR-β/-γ activator in platelets. However, other CCBs like amlodipine are PPAR-β activators, and lacidipine has no significant effect on PPARs activity in human platelets, suggesting that the activation of PPARs is not a common effect of all CCBs. Accordingly, the effects of different CCBs on platelet PPARs activity and the role of PPARs on CCBs-mediated antiplatelet activity are diverse and chemical structure specific. Upon activation by inducers such as collagen, a rapid release of PPAR-β/-γ from the α-granules of platelets into extracellular regions results in a marked reduction of the intracellular amount of PPAR-β/-γ, which may have a systemic effect. A novel finding is that nifedipine greatly inhibits the release of PPAR-β/-γ from activated platelets, thereby increasing the intracellular availability of PPAR-β/-γ which may enhance its cellular functions like the regulation of platelet activation. However, the underlying mechanisms accounting for the phenomenon require further investigation. Blocking PPAR-β/-γ activity with their specific antagonists significantly reverss the inhibitory effect of nifedipine on platelet aggregation, supporting that PPAR-β/-γ is involved in the antiplatelet activity of nifedipine. In addition, nifedipine-mediated up-regulation of the PI3K/Akt NO/cGMP/ PKG cascade that results in a reduction of Ca^2+^ mobilization is regulated by a PPAR-β/-γ-dependent signaling pathway (Figure 1). The activation of PKCα is crucial for platelet secretion and aggregation [24]. Consistent with our previous findings is the discovery that the inhibitory effect of PPAR-α/-γ on platelet PKCα activity is associated with its association with PKCα [50]. In collagen- stimulated platelets, nifedipine also induces an interaction between PPAR-β/-γ and PKCα accompanied by decreased PKCα activity evidenced by reduced PKCα phosphorylation in the complex. Similarly, an addition of PPAR-β/-γ antagonists abrogated the attenuation of PKCα activity by nifedipine, suggesting that direct interaction between PPAR-β/-γ and PKCα is a possible way to suppress PKCα activity.

Furthermore, administration of nifedipine markedly inhibited fluorescein sodium and irradiation-induced vessel thrombus formation *in vivo*. However, the antithrombotic effect of nifedipine was considerably reduced when PPAR-β/-γ antagonists were administrated simultaneously. Taken together, the antiplatelet and antithrombotic effects of nifedipine are mediated by activation of PPAR-β/-γ leading to up-regulation of the NO/cGMP/PKG cascade, as well as inhibition of PKCα activity and intracellular Ca^2+^ mobilization.

## NF-κB and platelet activation

NF-κB, a transcription factor, normally exists as an inactive cytoplasmic complex heterodimer complex composed of p50 and p65 subunits bound to the inhibitory protein, IκB-α. Upon stimulation, IκB-α is phosphorylated by IκB kinases (IKKs) leading to rapid degradation by proteasome and the subsequent release of NF-κB from its inhibitors. Then, the free NF-κB translocates to the nucleus, where it activates the transcription of inflammationrelated target genes [66]. The activation of the IKKβ/p65-NF- κB signaling pathway in human platelets is greatly amplified in response to thrombin or collagen. Blocking NF-κB activation by BAY11-7082 and Ro106-9920 inhibits platelet aggregation and granule release *via* the blockade of the ERK-cPLA_2_-TXA_2_ pathway, fibrinogen binding, platelet adhesion and, spreading in activated platelets [67]. Accordingly, suppressing NF-κB activation may be a potential target for inhibiting platelet aggregation. Previous studies have confirmed that the anti-inflammatory effect of PPAR-γ is associated with reducing NF-κB activation resulting from inhibiting IKKs in activated macrophages [68]. Therefore, the NF-κB activation is also regulated by PPARs. However, the effect of PPAR-γ on NF-κB activation is cell type and PPAR isoform specific [69].

**Fig. 3:**
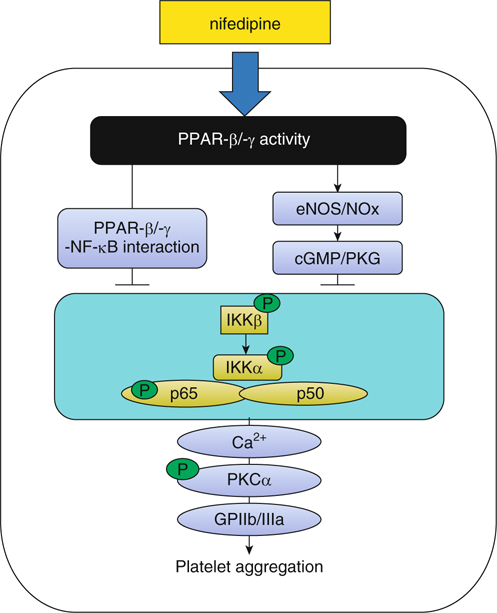
PPAR-β/-γ-dependent inhibition of NF-κB activation involves the antiplatelet activity of nifedipine. The decreased NF-κB activation accompanied by reduction of phosphorylation of IKK, IκBα, and p65NF-κB by nifedipine is mediated by a direct association of PPAR-β/-α with NF-κB and PPAR-β/ γ-dependent up-regulation of the NO/cyclic GMP/PKG1 pathway. This attenuates subsequent intracellular Ca^2+^ mobilization, PKCα activation, and surface GPIIb-IIIa expression, which in turn inhibits platelet activation.

## Antiplatelet activity of nifedipine is mediated by NF-κB activation *via* PPAR-β/-γ-dependent manner

Treatment with nifedipine decreass NF-κB activation by inhibiting IKK-β/IkBα phosphorylation in collagen-stimulated platelets. The inhibition of NF-κB activation is significantly reversed by specific PPAR-β/-γ antagonists, supporting the notion that PPAR-β/-γ negatively regulates NF-κB activation in platelets. Additionally, activation of NF-κB with betulinic acid (BetA) abolishes the nifedipine’s inhibition of intracellular Ca^2+^ mobilization and platelet aggregation, indicating that PPAR-β/-γ-mediated NF-κB activation involves the antiplatelet activity of nifedipine [70]. Notably, our research demonstrated for the first time that in activated platelets, nifedipine also induces an interaction of PPAR-β/-γ with NF-κB leading to decreased p65NF-κB phosphorylation in the complex. These results may provide a novel mechanism by which PPAR-β/-γ suppresses NF-κB activation in platelets through a direct interaction with NF-κB. Furthermore, the suppression of NF-κB activation by nifedipine is at least partly attributed to PPAR-β/-γ-dependent up-regulation of the NO/cyclic GMP/PKG1 pathway (Figure 3).

The binding of fibrinogen to the surface GPIIb/IIIa complex is a critical final step for platelet aggregation by crosslinking platelets and by the stabilization of aggregates. It has been reported that inhibiting NF-κB activation by NF-κB inhibitors reduces the outside-in/inside-out signaling of GPIIb/IIIa and fibrinogen binding in activated platelets [67]. Furthermore, GPIIb/IIIa is also required for NF-κB activation in human neutrophils [71], suggesting that there is a mutual activation between NF-κB and GPIIb/IIIa. Thus, the decreased surface GPIIb/IIIa expression by nifedipine may be a consequence of PPAR-β/-γ-down-regulated NF-κB activation as evidenced by blocking PPAR-β/-γ activity or enhancing NF-κB activation resulting in an elevated expression of GPIIb/IIIa.

In conclusion, nifedipine significantly increases the activity and intracellular levels of PPAR-β/-γ in activated platelets, which subsequently up-regulates the PI_3_K/Akt/eNOS/NO/cyclic GMP/ PKG cascade leading to the suppression of intracellular calcium mobilization, surface GPIIb/IIIa expression, and PKC activity *via* association of PPAR-β/γ with PKC. In addition, nifedipine is capable of inhibiting NF-κB activity through direct interaction of PPAR-β/-γ with NF-κB. These effects of nifedipine ultimately inhibit platelet aggregation and thrombosis formation. All told, nifedipine may be a potential drug for alleviating atherothrombosis and vascular diseases by targeting PPAR-β/γ-dependent signaling pathways in platelets.
